# Impact of aging on the tumor microenvironment in oral cancer: Mechanisms and therapeutic perspectives

**DOI:** 10.1016/j.jtumed.2026.09.008

**Published:** 2026-10-12

**Authors:** Prathibha Prasad, Ayman S. Amer, Anas Shamsi, Rashmi metgud, Khuzin Dinislam

**Affiliations:** a Basic Medical and Dental Sciences Department, College of Dentistry, Ajman University, Ajman, United Arab Emirates; b Center of Medical and Bio-allied Health Sciences Research, Ajman University, Ajman, United Arab Emirates; c Dept of Oral & Maxillofacial Pathology and Oral Microbiology, Pacific Dental College and Hospital, Pacific University, Rajasthan, India; d Department of General Chemistry, Bashkir State Medical University, Ufa, Russia

**Keywords:** الشيخوخة, أورام الفم, البيئة الدقيقة للورم, الشيخوخة الخلوية, تكوّن الأوعية الدموية, Aging, Angiogenesis, Cellular senescence, Mouth neoplasms, Tumor microenvironment

## Abstract

Oral cancer is a significant health problem worldwide, with particularly great impacts on elderly people. The aging process has a major impact on the tumor microenvironment (TME), affecting tumor initiation, progression, and the response to therapy. The present review summarizes the existing evidence regarding the effects of aging on the TME in oral cancer, the progression of oral diseases, and the implications for therapy. Studies were retrieved by conducting a structured literature search in the PubMed and MEDLINE databases from January 2010 to January 2025. Various age-related events such as cellular senescence, immunosenescence, extracellular matrix remodeling, chronic low-grade inflammation (inflammaging), and vascular dysfunction contribute to the creation of a pro-tumorigenic niche, which facilitates immune evasion and treatment resistance. The mechanisms of cellular senescence and immune deregulation, oxidative stress, and endothelial dysfunction are discussed in this review, as well as their roles in reducing anti-tumor immunity and therapeutic responses in elderly patients. Research shows that all of these age-related modifications are interconnected, and together they promote tumor progression and are involved in therapeutic resistance. Geriatric biology and TME research has highlighted new therapeutic approaches that could involve targeting senescence mechanisms, immune modulation, and vessel aging. Further insights into age-related TME dynamics are needed to develop aging-focused, tailored interventions that can enhance clinical results in older individuals with oral cancer.

## الملخص

يُعد سرطان الفم من المشكلات الصحية المهمة على مستوى العالم، وتزداد آثاره بصورة خاصة لدى كبار السن. وتؤثر عملية التقدم في العمر بشكل ملحوظ في البيئة الدقيقة للورم، بما ينعكس على بدء نشوء الورم وتطوره والاستجابة للعلاج. وتستعرض هذه المراجعة الأدلة المتاحة حول تأثيرات الشيخوخة في البيئة الدقيقة للورم في سرطان الفم، وتطور أمراض الفم، وانعكاسات ذلك على العلاج.

تم جمع الدراسات من خلال بحث منهجي في الأدبيات المنشورة ضمن قاعدتي بيانات PubMed وMEDLINE خلال الفترة من يناير 2010 إلى يناير 2025. وتسهم عدة تغيرات مرتبطة بالتقدم في العمر، مثل الشيخوخة الخلوية، وشيخوخة الجهاز المناعي، وإعادة تشكيل المصفوفة خارج الخلوية، والالتهاب المزمن منخفض الدرجة المرتبط بالشيخوخة، واضطراب وظائف الأوعية الدموية، في تكوين بيئة مواتية لنمو الورم، مما يساعد على الإفلات من الاستجابة المناعية ويزيد من مقاومة العلاج.

وتناقش هذه المراجعة آليات الشيخوخة الخلوية، واضطراب تنظيم الجهاز المناعي، والإجهاد التأكسدي، والخلل الوظيفي في بطانة الأوعية الدموية، إضافة إلى أدوارها في إضعاف المناعة المضادة للأورام وتقليل الاستجابة للعلاج لدى المرضى كبار السن. وتشير الأبحاث إلى أن هذه التغيرات المرتبطة بالعمر مترابطة فيما بينها، وأنها تعمل مجتمعة على تعزيز تطور الورم والمساهمة في مقاومة العلاج.

وقد أسهمت الأبحاث في مجال بيولوجيا الشيخوخة والبيئة الدقيقة للورم في إبراز توجهات علاجية جديدة قد تشمل استهداف آليات الشيخوخة الخلوية، وتعديل الاستجابة المناعية، واستهداف شيخوخة الأوعية الدموية. ولا تزال هناك حاجة إلى فهم أعمق للتغيرات المرتبطة بالعمر في البيئة الدقيقة للورم من أجل تطوير تدخلات علاجية مخصصة تراعي التقدم في العمر، بما يسهم في تحسين النتائج السريرية لدى كبار السن المصابين بسرطان الفم.

## Introduction

Oral cancer is a major global health challenge that most commonly presents as oral squamous cell carcinoma (OSCC), which accounts for more than 90% of cases. Despite advances in diagnosis and treatment, many patients present at advanced stages, resulting in poor survival outcomes.^1^ According to GLOBOCAN 2020, oral cancer accounts for approximately 377,000 new cases and 177,000 deaths annually worldwide, with a particularly high burden in South Asia, which is largely attributable to tobacco use, betel nut consumption, and alcohol exposure.^2^ Additional risk factors include human papillomavirus infection, chronic inflammation, periodontitis, radiation exposure, and nutritional deficiencies. The disease predominates in males and the 5-year survival rate is ∼50%.^3^

Aging is characterized by the progressive loss of physiological integrity driven by interconnected biological processes, including genomic instability, telomere attrition, epigenetic alterations, mitochondrial dysfunction, impaired proteostasis, and cellular senescence.^4^ These hallmarks of aging promote systemic immune decline, chronic low-grade inflammation (inflammaging), and reduced regenerative capacity, all of which increase the susceptibility to cancer.^5^ Importantly, aging reshapes tissue homeostasis and alters the local microenvironment in ways that favor tumor initiation and progression.^6^

The tumor microenvironment (TME) comprises malignant epithelial cells, cancer-associated fibroblasts (CAFs), immune infiltrates, endothelial cells, extracellular matrix (ECM) components, and soluble mediators.^7^ Rather than being a passive scaffold, the TME actively regulates tumor growth, invasion, immune evasion, and therapeutic response. Age-associated alterations, including the accumulation of senescent stromal cells, fibrosis, ECM stiffening, immune dysregulation, telomere attrition, and vascular dysfunction, contribute to the formation of a pro-tumorigenic niche.^8^^,^^9^

In the oral cavity, lifelong exposure to carcinogens such as tobacco, alcohol, and betel nut, as well as chronic mechanical irritation and microbial shifts, further compound age-related tissue changes. Aging of the oral mucosa is associated with fibroblast senescence, altered ECM composition, and changes in the oral microbiome, which collectively enhance oxidative stress and sustain chronic inflammation.^10^ These factors lead to a permissive environment that supports malignant transformation and tumor progression.

This review discusses the relationships between aging and oral cancer TME and its cellular components, that is, cellular senescence, immune aging, chronic inflammation, ECM remodeling, tumor-associated fibroblasts, and vascular dysfunction. The aim is to identify mechanistic connections with geriatric biology and to present possible therapeutic approaches for elderly patients by combining geriatric biology and TME research.

A structured literature search was performed in the PubMed and MEDLINE databases to retrieve studies focused on aging, the TME, and oral cancer. Articles published between January 2010 and January 2025 were included. The following key words and Boolean operators were used: “Aging,” “Tumor Microenvironment,” “Oral Cancer,” “Cellular Senescence,” and “Chronic Inflammation.”

Included studies were randomized controlled trials, cohort studies, case–control studies, observational studies, or systematic reviews that assessed the effects of aging on the TME in oral cancer. Selected articles were also searched manually to find more relevant articles. Conceptual clarity was essential and achieved by using authoritative textbooks and peer-reviewed journal articles when needed.

Studies without available full text or that only appeared as abstracts were excluded. Conference proceedings, letters to the editor, opinion-based publications, and studies addressing cancers other than oral cancer were also excluded.

Peer-reviewed English-language original studies and systematic reviews relevant to aging and the TME in oral cancer were considered. Studies that evaluated factors unrelated to aging were excluded.

## Aging and the TME: an overview

The TME is a dynamic and interactive network composed of malignant cells, stromal components (fibroblasts, immune cells, and endothelial cells), soluble mediators, and ECM. Instead of serving as a passive scaffold, the TME actively regulates tumor initiation, progression, immune evasion, and therapeutic resistance.^11^ Functionally, the TME can be considered as comprising several interconnected niches, including hypoxic, metabolic, immune, mechanical, and neural microenvironments.^12^^,^^13^ Rapid tumor proliferation combined with an inadequate vascular supply results in regional hypoxia. Hypoxia-inducible factor-1 (HIF-1) mediates cellular adaptation to low oxygen tension by promoting glycolysis and suppressing oxidative phosphorylation, which leads to a build-up of lactate, lowering the extracellular pH, increasing the aggressiveness of tumors, immune suppression, and resistance to therapy.^14^^,^^15^

Another major characteristic of the TME is chronic inflammation. Pro-tumorigenic inflammatory responses that regulate signaling pathways such as MAPK, JAK-STAT, and NF-κB promote the growth and spread of tumors. In addition, abnormal tumor blood vessels contribute to immune evasion and impaired drug delivery, further supporting disease progression.^16^

The TME changes substantially with aging. One of the most important changes that occur with aging is due to the development of the senescence-associated secretory phenotype (SASP), where senescent stromal cells produce pro-inflammatory cytokines, chemokines, and matrix remodeling enzymes. Cellular senescence is initially a tumor suppressive mechanism, but ultimately leads to chronic SASP. The interaction of tumor cells with the inflammatory microenvironment is known to support tumor progression and immune escape.^17^

Immunosenescence further modifies the aged TME. Age-related decline in antigen presentation, the reduced cytotoxic activities of T lymphocytes and natural killer cells, and expansion of immunosuppressive populations such as regulatory T cells (Tregs) and myeloid-derived suppressor cells (MDSCs) collectively impair anti-tumor immunity.^18^ Concurrent vascular dysfunction and endothelial impairment exacerbate hypoxia and compromise therapeutic efficacy.^19^ Evidence from multiple malignancies supports the influence of aging on tumor biology, where age-associated oxidative stress-mediated ECM remodeling enhances metastasis in lung cancer,^20^ immunosuppression accelerates progression in prostate cancer, and vascular alterations reduce the treatment efficacy in glioblastoma. In particular, in oral cancer, hypoxia, oxidative stress, and chronic inflammation contribute to chromosomal instability, malignant transformation, and resistance to therapy.^21^

## Cellular senescence and the aged TME in oral cancer

Cellular senescence is a state of stable cell cycle arrest that differs from quiescence, differentiation, or apoptosis. It may arise from replicative exhaustion due to telomere shortening or from stress-induced premature senescence triggered by oxidative damage, irradiation, or oncogenic signaling.^22^ Senescence is initially tumor-suppressive, but exerts paradoxical pro-tumorigenic effects through the SASP.^23^

SASP consists of pro-inflammatory cytokines, growth factors, proteases, and extracellular vesicles that accumulate during aging and contribute to chronic low-grade inflammation. In the oral cavity, persistent SASP signaling disrupts immune homeostasis, promotes stromal remodeling, and facilitates malignant transformation (Figure 1). Thus, senescence represents a dual-edged mechanism that prevents uncontrolled proliferation in the early stages but that later fosters tumor progression through chronic inflammatory signaling.^24^

**Figure 1:**
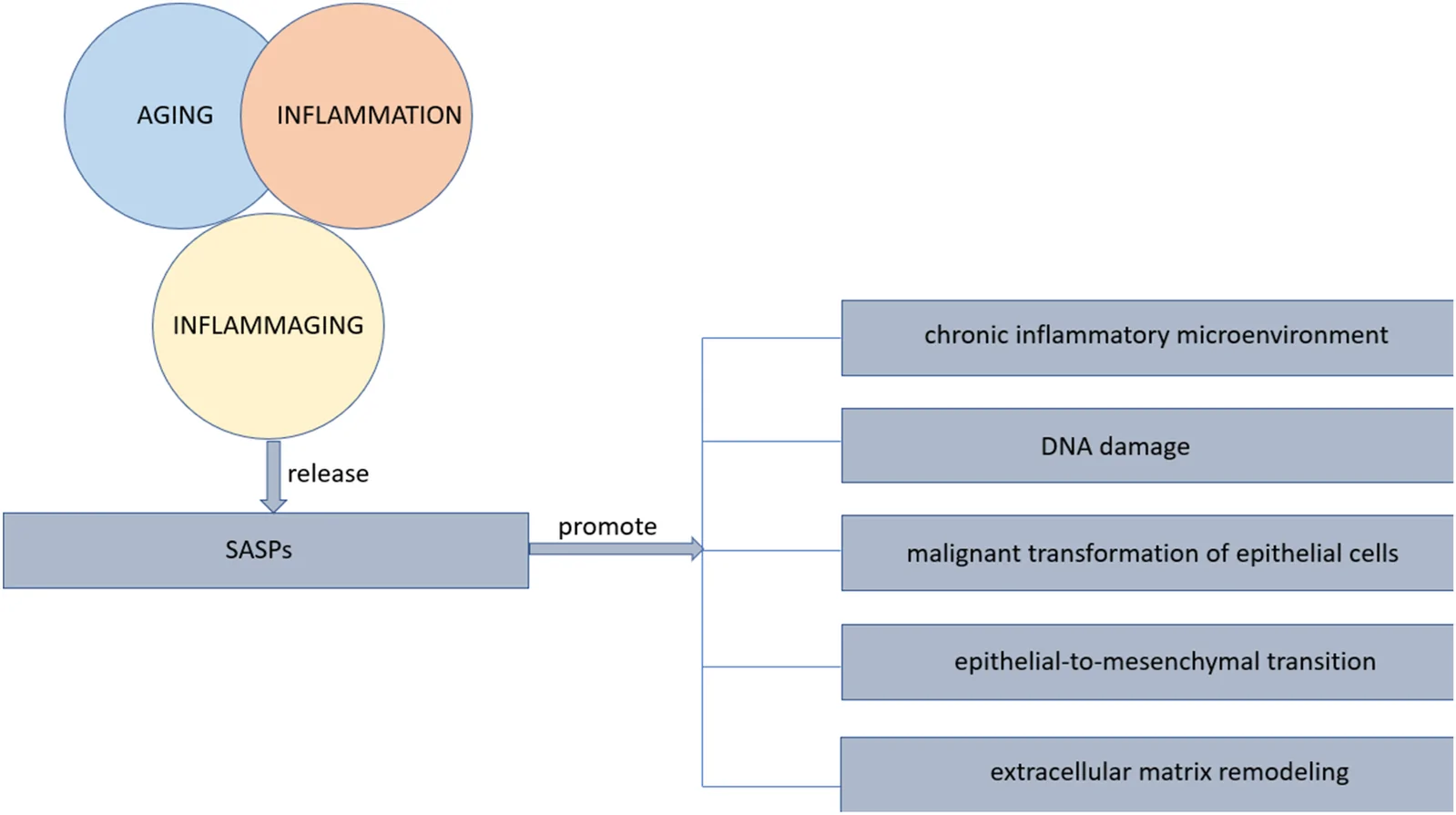
A diagram showing the interactions between aging, inflammation, and inflammaging that drive cancer progression. Aging and chronic inflammation converge to drive inflammaging, characterized by the release of SASP factors. SASP promotes a chronic inflammatory microenvironment, DNA damage, malignant transformation of epithelial cells, epithelial-to-mesenchymal transition, and extracellular matrix (ECM) remodeling, which collectively enhance tumor progression and invasiveness.

The shortening of telomeres is a key mechanism related to replicative senescence. As the telomeres shorten over time during cell division, the DNA damage response and mitochondrial dysfunction are triggered to ultimately cause senescence. Genomic instability is another characteristic of oral carcinogenesis that can be caused by critically short telomeres. In established cancers, the reactivation of telomerase allows cancer cells to proliferate even though the telomeres are eroding. Genetic changes, including GSTM1 polymorphism, have been linked to changes in the telomeres in oral cancer, indicating that there may be a relationship between susceptibility to oxidative stress and chromosomal instability.^25^

## Immune system aging and the oral cancer microenvironment

Anti-tumor immunity is altered considerably by aging, and this is reflected by immunosenescence, which involves reductions in the activities of CD8^+^ T cells and natural killer cells, and increases in Tregs and MDSCs, which contribute to an immunosuppressive TME. Immune checkpoint molecules are also upregulated, including PD-1 and CTLA-4, which further dampen T-cell activation.^26^

In the elderly, tumor-associated macrophages predominantly have the M2 phenotype with pro-angiogenic and pro-tumor activities rather than anti-tumor activity. These coordinated immune changes create a "permissive" environment for the development of OSCC.^27^

Intriguingly, emerging evidence suggests that older people might still benefit from immune checkpoint inhibition despite immune deficits, indicating the need for personalized immunotherapeutic approaches in aging populations.^28^

## Chronic inflammation in aging-associated oral cancer

Persistent low-grade systemic inflammation or “inflammaging” is a hallmark of aging that plays an important role in the onset of oral cancer. Neurodegenerative and chronic inflammatory diseases like Parkinson's disease and Oral Lichen Planus (OLP)s are characterized by a pro-tumorigenic microenvironment, which involves prolonged cytokine production and oxidative stress.^29^

High concentrations of TNF-α, IL-6, and IL-8 induce oncogenic processes such as the NF-κB signaling pathway and promote tumor cell survival, invasion, and ECM degradation.^30^ In addition, immune regulation and metabolic changes in aging further support an immunosuppressive microenvironment that encourages tumor progression.^31^

## ECM remodeling in aging and oral cancer

The oral mucosa comprises stratified squamous epithelium and connective tissue, and changes in the ECM occur during aging.^32^ Tumor initiation and behavior are greatly affected by the composition and mechanical properties of the ECM. The ECM controls tumor cell adhesion, migration, and proliferation. ECM interactions with integrin drive tumor cell behavior, and the integrin-mediated signaling pathways include FAK/Src and MAPK.^33^ Age-associated matrix stiffening enhances mechanotransduction, promoting invasive behavior and resistance to apoptosis.

Receptors for hyaluronan (HA), including CD44 and TLR4, are known to activate proliferative and inflammatory pathways, including EGFR and NF-κB signaling. HA is often overexpressed in oral cancers and has been linked to decreased therapeutic responsiveness.^34^

Heparan sulfate proteoglycans such as syndecan-1 promote tumor progression, bind growth factors, and can remodel the ECM. Tenascin-C disrupts normal cell–matrix adhesion, promoting growth and angiogenesis.^35^

Proteoglycans control cell–matrix interactions, growth factor signaling, and inflammatory responses to regulate the oral cancer microenvironment. The transmembrane heparan sulfate proteoglycan syndecan-1 is often modified during the course of OSCC progression and plays a role in tumor invasion by modulating ECM organization, fibroblast activation, and signaling pathways involving growth factors and cytokines. Alterations in the turnover or loss of ECM-related proteoglycans may promote the establishment of an environment that is conducive to malignant transformation and the loss of tissue homeostasis.

Matrix metalloproteinases (MMPs) are important enzymes that mediate the degradation and remodeling of the ECM during oral carcinogenesis. MMP-1, MMP-2, and MMP-13 are highly expressed in OSCC and are linked to enhanced tumor invasion, metastasis, and poor clinical outcomes.

The mechanotransduction axis connects changes in the ECM associated with aging to malignant progression by activating Hippo pathway effectors YAP and TAZ.^36^

Tumor invasion is also promoted by MMPs, which act by degrading ECM molecules and regulating cytokines and growth factors. Elevated expression levels of MMP-1, MMP-2, and MMP-13 are associated with poor prognosis and malignant transformation in oral cancer.^37^

## ECM remodeling and metastasis in aging-associated oral cancer

ECM remodeling is a critical determinant of invasion and metastasis in OSCC.^38^ In collagen-rich and mechanically stiffened matrices, aging tumor cells are exposed to sustained biomechanical stress and pro-invasive signaling cues. Figure 2 summarizes the roles of the epithelial–mesenchymal transition (EMT) in oral cancer, which involves resistance to anoikis, acquisition of motility, degradation of the surrounding matrix, and intravasation into the vasculature.^39^

**Figure 2:**
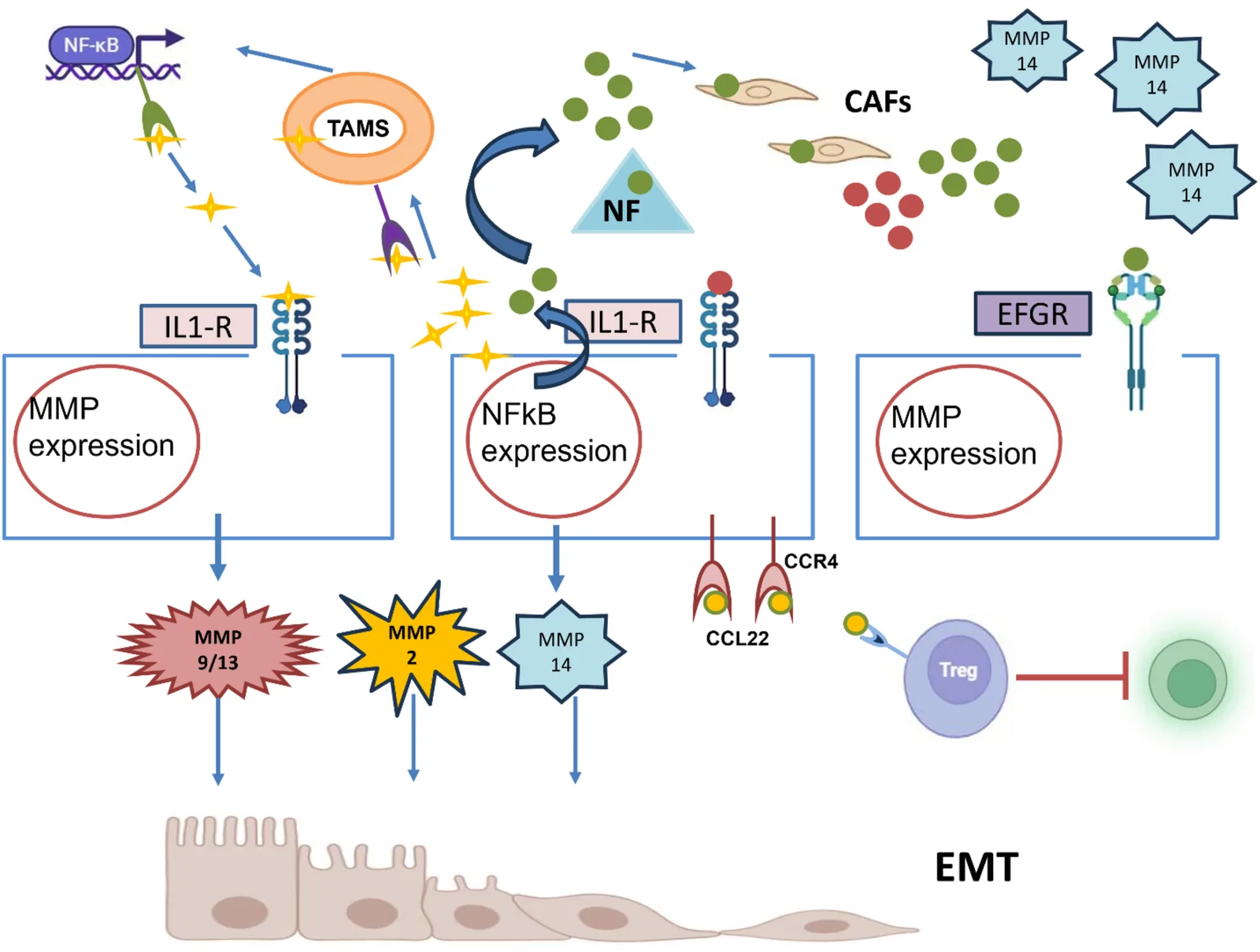
Epithelial-to-mesenchymal transition (EMT). Schematic representation of the interaction between tumor cells and the microenvironment that causes oral cancer to progress.

A common mechanism that mediates these processes is the EMT, which is defined by the downregulation of epithelial markers such as E-cadherin and upregulation of mesenchymal transcription factors such as SNAIL and TWIST.^40^

Age-related stiffening of the ECM also leads to an increase in ECM-mediated signaling through the integrin-FAK/Src pathway, promoting EMT and the invasive behavior of tumor cells.^41^ Mechanotransduction pathways such as YAP/TAZ further allow tumor cells to adapt to stiffening of the ECM and to continue proliferating under mechanical stress.^42^

Transforming growth factor-β (TGF-β) is a major inducer of EMT. In the aging microenvironment, persistent TGF-β signaling contributes to fibrosis, stromal activation, and metastatic progression.^43^ Moreover, changes in the composition of ECM and these cellular signals lead to the establishment of an environment that supports the dissemination of tumor cells. The increased ECM stiffness enhances the clustering of integrin and activates downstream FAK/Src signaling, thereby increasing cytoskeletal remodeling, migration, and the invasion of oral cancer cells. In addition, YAP/TAZ activation in response to mechanical stress controls transcriptional programs related to the survival of tumor cells, growth, and the ability to invade and spread.

Oral cancer metastasis is another important step in ECM degradation and is mostly mediated by MMPs. MMPs promote invasion by degrading the basement membrane, allowing tumor cells to migrate into the components and interstitial ECM proteins in surrounding tissues, and to access the vasculature. In addition, MMPs release matrix-bound growth factors to regulate the TME by modifying cytokine signaling, further enhancing the angiogenesis process, inflammation, and immune evasion. Age-related accumulation of senescent stromal fibroblast cells, chronic inflammation, and activation may enhance the expression and activity of MMPs, thereby promoting ECM remodeling and metastasis.

Thus, the interplay between ECM stiffening, EMT activation, proteolysis, and epithelial and stromal alterations are major mechanisms during aging that lead to aggressive oral cancer behavior in the TME.

## Aging and tumor-associated fibroblasts in oral cancer

The most abundant stromal cells in the oral mucosa are the fibroblasts, which are crucial for tissue repair and the maintenance of matrix homeostasis.^44^ In cancer, activated fibroblasts (also known as CAFs) have a pro-tumorigenic phenotype, where they express fibroblast activation protein and alpha smooth muscle actin.^45^

Aging is also known to promote the accumulation of senescent and activated fibroblasts called myofibroblastic CAFs, which secrete excessive amounts of ECM components and their abundance is known to be increased in advanced oral lesions.^46^^,^^47^ Their expansion is correlated with tumor progression.

CAFs also have major immunomodulatory impacts, where they secrete cytokines, including CXCL12 and TGF-β, which attract immunosuppressive cells including tumor-associated macrophages, MDSCs, and Tregs, and limit anti-tumor immunity.^48^ Therefore, age-related stromal activation may amplify immune evasion in OSCC.

Emerging strategies aim to reprogram rather than eliminate CAFs by targeting pathways such as TGF-β, IL-6, and CXCL12/CXCR4 signaling to reduce desmoplasia and restore immune responsiveness.^49^ However, given the heterogeneity of CAFs, therapeutic modulation requires careful contextual evaluation.

## Angiogenesis, endothelial dysfunction, and aging

Neovascularization is necessary when the growth of a tumor exceeds a certain size. However, tumor-associated blood vessels are structurally abnormal, poorly organized and hyperpermeable, thereby leading to poor oxygen and drug delivery, which are further worsened by aging and aging-associated endothelial dysfunction causing increased vascular permeability, chronic inflammation, and impaired immune cell trafficking.^50^

Existing approaches are focused on vascular normalization to promote oxygenation and increase the effectiveness of therapeutic agents, rather than the complete inhibition of angiogenesis. Thus, age-induced changes in the vasculature have both pro- and anti-tumor promoting effects under treatment.

## Hypoxia in the aging TME

Hypoxia is a hallmark of the TME and is exacerbated by the abnormal blood supply to the tumor.^51^ Hypoxia contributes to the production of angiogenic factors, metabolic reprogramming, vasculogenic mimicry and intercellular communication via exosomes in OSCC.^52^

Aging tissue may be characterized by many disrupted blood vessels and chronic inflammation, thereby increasing hypoxic stress, and thus the aggressiveness and therapeutic resistance of tumors in these tissues.

## Therapeutic implications for the aging population

Oral cancer treatment in seniors is fraught with challenges. Treatment decisions are complicated by age-related comorbidities, frailty, and immunosenescence, and the treatment should be tailored to the individual.

## Chemotherapy and aging

Chemotherapy is an integral part of OSCC treatment, but it can increase age-related vulnerabilities. The use of cytotoxic agents may lead to therapy-induced senescence of stromal and immune cells, leading to chronic inflammation and tumor recurrence. Aging microenvironments may also facilitate chemoresistance by increasing cell–matrix adhesion and signaling to promote immunosuppression.^24^

## Targeting the TME

Given the central role of the TME in aging-associated tumor progression, therapeutic strategies increasingly target stromal and microenvironmental components, as follows.

• **ECM modulation:** Angiotensin receptor blockers (e.g., losartan) reduce TGF-β–mediated fibrosis and enhance drug delivery.^53^
• **MMP inhibition:** Selective targeting of MMPs aims to limit invasion and metastasis.^54^
• **Vascular targeting:** Clinical efficacy of anti-VEGF treatments has been shown in some cancers, but with different degrees of effectiveness.^55^
• **Hypoxia and acidosis modulation:** Inhibition of proton transporters and carbonic anhydrases aims to reverse acidification in tumors.^56^
• **Immune modulation:** CSF-1R signaling or inflammatory cytokines (IL-1) are targets of strategies to reverse immunosuppression within the TME.^57^

## Future perspectives and challenges

### Nanotechnology and targeted delivery

Nanomedicine can be applied in promising strategies for targeting the aged TME. Functionalization of nanoparticles with ligands such as arginine–glycine–aspartic acid (RGD) peptides enables selective binding to integrin receptors, enhancing tumor-specific delivery.^58^ Multifunctional nanocarriers capable of co-delivering chemotherapeutic agents and microenvironment-modulating compounds may improve the therapeutic efficacy while also reducing systemic toxicity. However, clinical translation remains challenging due to variability in tumor biology, pharmacokinetics, and long-term safety concerns. Future research should prioritize age-specific modeling of nanotherapeutic responses.

### Improved preclinical models

Despite advances in molecular classification, the treatment of oral carcinoma remains largely stage based. Patients within the same stage frequently receive similar therapeutic regimens, irrespective of biological heterogeneity. Developing preclinical models that accurately recapitulate molecular subgroups of head and neck carcinomas, including age-associated alterations in the TME, will be essential for translating molecular insights into clinical benefit. Age-relevant experimental systems are particularly necessary to understand how immunosenescence, stromal activation, and vascular dysfunction influence the therapeutic response.

### Multiomics and precision medicine

Integrating genomics, transcriptomics, proteomics, and metabolomics (“multiomics”) can potentially refine risk stratification and identify predictive biomarkers for therapy selection. A combination of molecular profiling and geriatric assessment could allow the development of more precise and personalized treatment strategies according to aging. However, there are still some challenges because the TME is complex and heterogeneous among patients. Intrinsic resistance mechanisms and adaptive resistance might reduce the effectiveness of TME-targeted treatments. Furthermore, the TME is also modified by conventional treatments such as chemotherapy and radiotherapy, and sometimes in a pro-survival or immunosuppressive manner. Understanding these dynamic interactions in greater detail will be important for optimizing therapeutic sequencing and combination approaches.

## Conclusion

Aging is the core process that affects the oral cancer microenvironment by reshaping the environment to provide suitable conditions for tumor initiation, progression, immune escape, and therapeutic resistance. All of these processes, including immunosenescence, chronic inflammation, ECM remodeling, vascular dysfunction, and the accumulation of senescent stromal cells, lead to the formation of a pro-tumorigenic niche in elderly patients. It is important to understand the aging process as a biological modifier rather than as a simple chronological variable to better understand how to improve the outcome of oral cancer.

Future research should aim to establish age-appropriate experimental models that reflect the complex interplay between tumor cells and the aged TME. Integrating multiomics approaches, longitudinal clinical studies, and molecular profiling is important for identifying age-associated biomarkers and validating potential therapeutic targets. Studies should also investigate the effects of aging on the responses to combination treatments and targeted therapies, as well as immunotherapy. Understanding the changes in the TME associated with aging will be essential for progress in precision medicine, and age-adapted treatments and interventions in order to improve survival outcomes and the quality of life for older people with oral cancer.

## Author contributions

All authors contributed to the study conception and design. PP performed data collection and analysis. The first draft of the manuscript was written by PP, AS, and ASA. All authors commented on previous versions of the manuscript. DK and RM critically revised the manuscript. All authors read and approved the final version.

## Data availability

All relevant data are included within the manuscript.

## Ethics approval

Not applicable.

## Consent to participate

Not applicable.

## Consent for publication

Not applicable.

## Source of funding

The authors acknowledge the Deanship of Research and Graduate Studies at 10.13039/501100019286Ajman University, United Arab Emirates for supporting this publication.

## Conflict of interest

The authors have no competing interests to declare.
